# Molecular mechanism of histone variant H2A.B on stability and assembly of nucleosome and chromatin structures

**DOI:** 10.1186/s13072-020-00351-x

**Published:** 2020-07-14

**Authors:** Junhui Peng, Chuang Yuan, Xinfan Hua, Zhiyong Zhang

**Affiliations:** 1 MOE Key Laboratory for Membraneless Organelles & Cellular Dynamics, National Science Center for Physical Sciences at Microscale, School of Life Sciences, University of Science and Technology of China, Hefei, Anhui 230026 People’s Republic of China; 2 Present Address: Laboratory of Evolutionary Genetics and Genomics, The Rockefeller University, New York, NY 10065 USA

**Keywords:** Histone variant H2A.B, Multiscale modeling, Molecular dynamics, Coarse-grained simulations

## Abstract

**Background:**

H2A.B, the most divergent histone variant of H2A, can significantly modulate nucleosome and chromatin structures. However, the related structural details and the underlying mechanism remain elusive to date. In this work, we built atomic models of the H2A.B-containing nucleosome core particle (NCP), chromatosome, and chromatin fiber. Multiscale modeling including all-atom molecular dynamics and coarse-grained simulations were then carried out for these systems.

**Results:**

It is found that sequence differences at the C-terminal tail, the docking domain, and the L2 loop, between H2A.B and H2A are directly responsible for the DNA unwrapping in the H2A.B NCP, whereas the N-terminus of H2A.B may somewhat compensate for the aforementioned unwrapping effect. The assembly of the H2A.B NCP is more difficult than that of the H2A NCP. H2A.B may also modulate the interactions of H1 with both the NCP and the linker DNA and could further affect the higher-order structure of the chromatin fiber.

**Conclusions:**

The results agree with the experimental results and may shed new light on the biological function of H2A.B. Multiscale modeling may be a valuable tool for investigating structure and dynamics of the nucleosome and the chromatin induced by various histone variants.

## Background

In the nucleus of eukaryotic cells, DNA is usually in the form of highly folded chromatin. The nucleosome is the basic structural unit of the chromatin, which consists of the core particle and the linker DNA [1]. It is known that a nucleosome core particle (NCP) is composed of an octamer of canonical histones, including two copies each of H2A, H2B, H3, and H4 and approximately 146 base pairs of DNA wrapped around the octamer [2]. The NCPs are connected by linker DNAs that typically range from 10 to 90 base pairs to form a nucleosomal array [3]. A linker histone (H1 or H5) binds to the linker DNAs near the DNA entry and exit sites of each NCP that is called a chromatosome [4], which would fold the nucleosomal array into a condensed fiber [5]. These different levels of DNA compaction play important roles in all biological processes involving DNA, such as gene transcription.

There are a number of ways to regulate the dynamic mode of nucleosome and chromatin, and one of them is to replace the canonical histones with histone variants. A histone variant has a more or less different amino acid sequence than the corresponding canonical histone, which may change the structure and dynamics of the nucleosome and the chromatin fiber [6]. Among all the canonical histones, H2A has the largest number of variants, such as H2A.X, H2A.Z, macroH2A, and H2A.B, which play diverse functional roles [7].

**Fig. 1:**
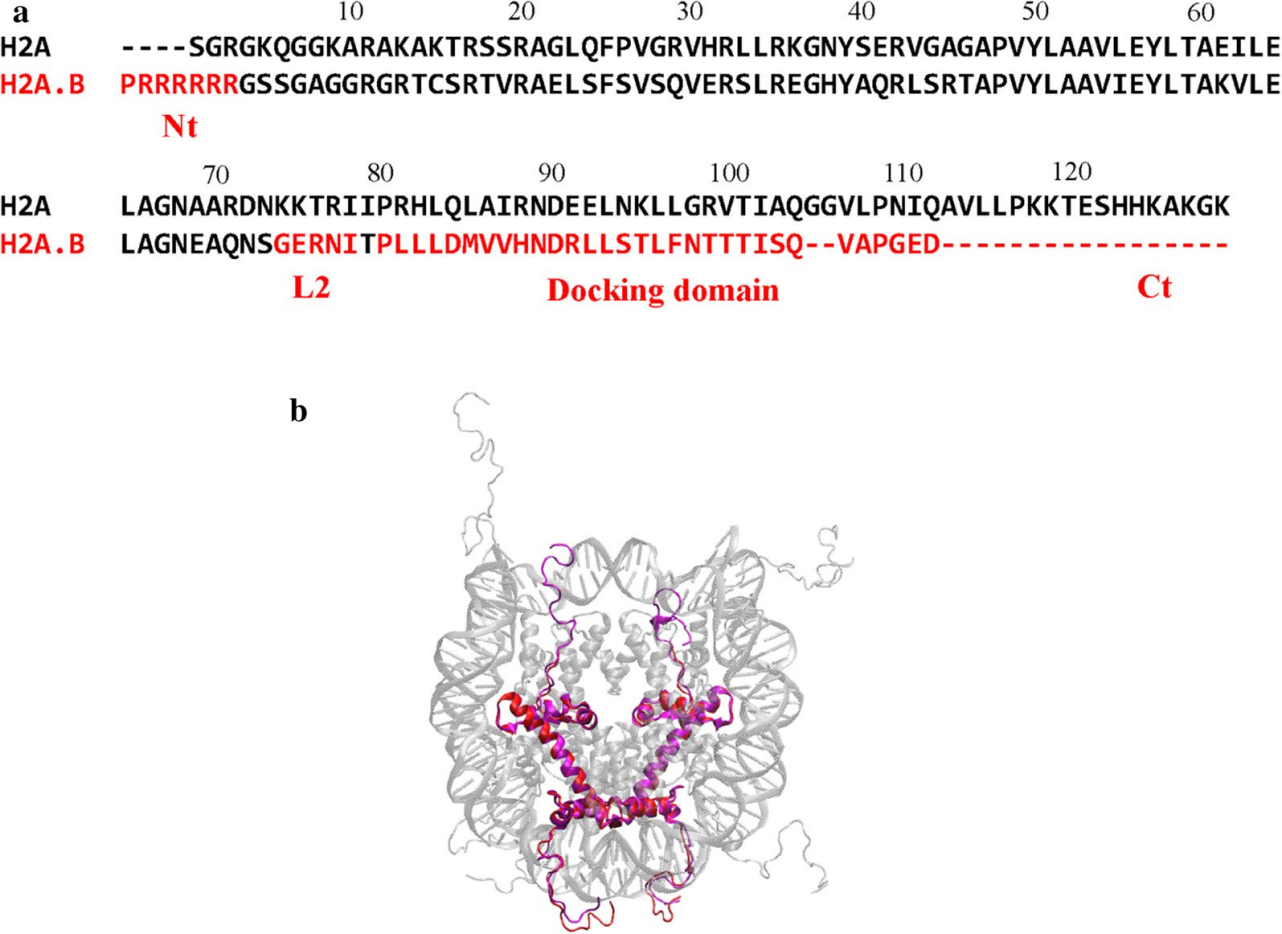
Comparison between H2A and H2A.B. **a** Sequence alignment of the human H2A and H2A.B. These different regions including the C-terminal tail (Ct), the docking domain, the L2, and the N-terminal tail (Nt) are labeled. **b** Structural alignment of the H2A NCP and the predicted H2A.B NCP in which the two copies of H2A.B are colored in red and H2A are colored in magenta

H2A.B, also called H2A.Bbd (Barr body deficient), is an unusual variant whose sequence identity is only 48% compared to the canonical H2A and is associated with active gene transcription [8]. The major sequence differences between H2A.B and H2A are shown in Fig. 1a: (1) the C-terminal tail (Ct) and the last segment of the docking domain are missing in H2A.B, (2) the L2 loop in H2A.B is more basic than that in H2A, and (3) the N-terminal tail (Nt) of H2A.B contains a continuous stretch of six arginines. These differences may alter the interactions within the NCP and change its structure and dynamic properties. The ability of the H2A.B-containing NCP (denoted as the H2A.B NCP) to interact with the linker histone may also be modulated, which could affect the higher-order structure of the chromatin. Biochemical and biophysical studies reveal that the H2A.B NCP is more extended than the H2A NCP because the DNA regions at the entry/exit sites are flexibly detached from the octamer in the former [9–12]. However, to the best of our knowledge, a high-resolution structure of the H2A.B NCP is still lacking except for a crystal structure of the H2A.B–H2B dimer [13]. Therefore, the underlying molecular mechanism of this histone variant in regulating the stability and assembly of nucleosome and chromatin remains elusive.

The above question could rely on computational studies that complement experiments to provide a detailed molecular view of the problem [14, 15]. For such studies, one can choose either atomistic or coarse-grained (CG) simulations. The former, such as the all-atom molecular dynamics (AA-MD) simulation, is proven to be a valuable tool to investigate the conformational dynamics of large biomolecules at a fine resolution [16], and hardware and software advances have allowed us to simulate ever more complex biomolecules [17]. However, AA-MD simulations are computationally demanding and can only sample a limited conformational space at the current accessible time scale of microseconds, thereby raising the issue of convergence. CG methods, on the other hand, have been popularly used to study larger length-scale biomolecules at longer time scales (milliseconds or longer) than AA-MD [18–20]. CG simulations can achieve equilibration quickly, but at the cost of missing atomic details.

In this work, we present a multiscale computational study [21, 22] on H2A.B NCP using both AA-MD and CG simulations. These two techniques are complementary to each other. AA-MD simulations in explicit solvent probe structural dynamics and specific interactions in atomic detail, whereas CG simulations can sample conformational space more extensively and explore more global properties of the NCP. In this way, we may cross-validate the results that are obtained from the two independent methods. In addition, CG simulations on the H2A.B-containing chromatosome and fiber are conducted. Based on the simulation data, we observe conformational changes of the H2A.B NCP, chromatosome and fiber that are consistent with the experimental studies, and then the relevant molecular mechanism of this histone variant can be addressed.

## Results

### DNA is more relaxed in the H2A.B NCP

Using the H2A NCP structure as the template, we built a structural model of H2A.B NCP (Fig. 1b) using homology modeling [23]. Multiscale simulations were then conducted for the both systems.

**Fig. 2:**
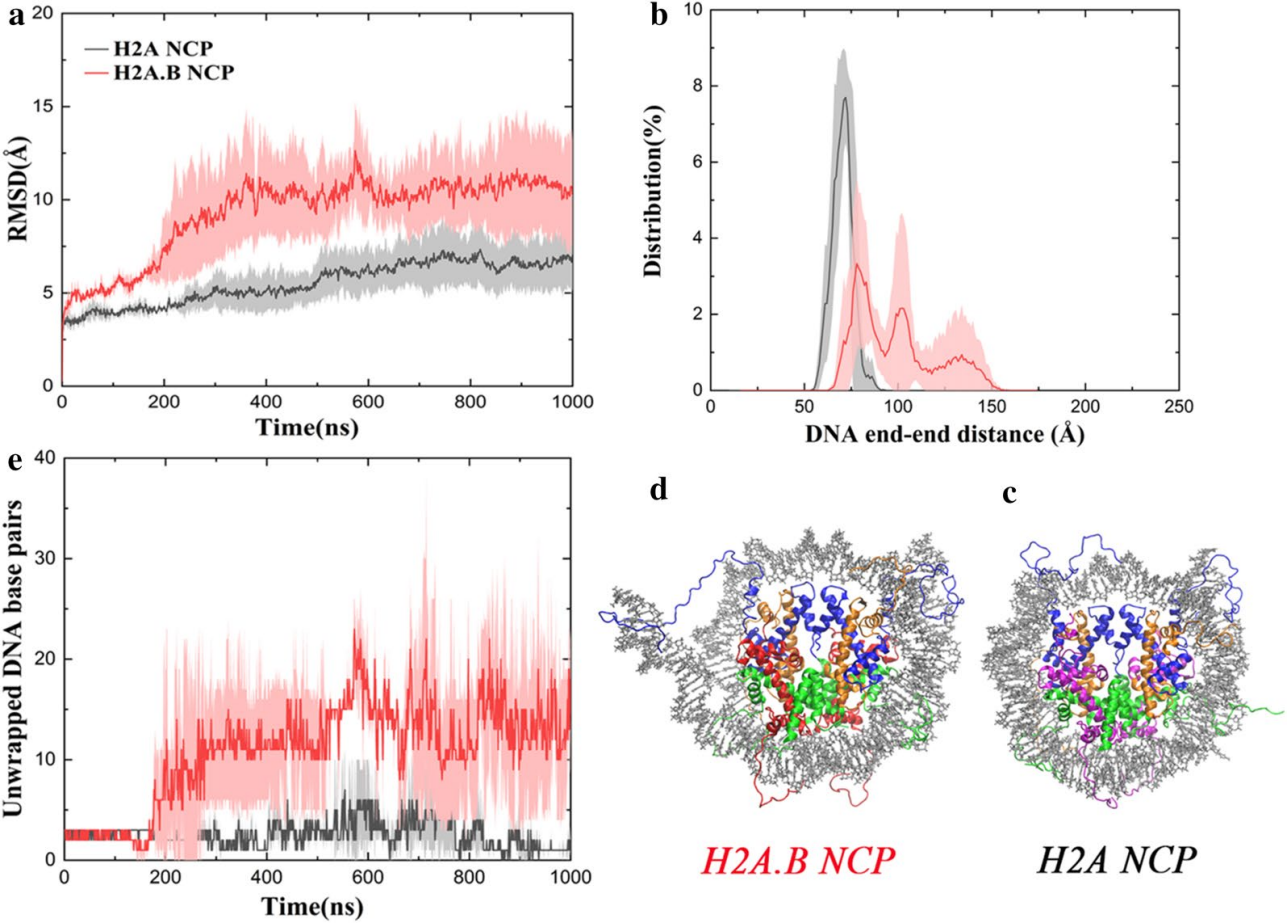
The H2A.B NCP is more dynamic than the H2A NCP. **a** Time evolution of the RMSD of the DNA during the AA-MD simulations. The RMSD values were calculated using all the P atoms. **b** Distribution of the DNA end-to-end distances from the AA-MD simulations. The distances were calculated between the first P atom at the entry and the first P atom at the exit. **c** A representative structure of the H2A NCP that has a peak DNA end-to-end distance of 72 Å. **d** A representative structure of the H2A.B NCP that has a peak DNA end-to-end distance of 128 Å. **e** Time evolution of the number of the unwrapped DNA base pairs during the MD simulations. In **a**, **b**, **e** for each system, average values calculated from the three independent simulations are plotted, and standard deviations are shown as errors that are represented by shade

Experimental data have shown that the H2A.B NCP has different properties from the H2A NCP, and, among them, the most important feature is the highly dynamic DNA in the H2A.B NCP [9–12]. To show the conformational changes of the DNA in the MD simulations, we calculated the root mean square deviations (RMSDs) of the DNA in the H2A and the H2A.B NCP using all the P atoms (Fig. 2a). During the MD simulations, the DNA in the H2A NCP is relatively stable with an average RMSD of 7.0 ± 1.5 Å (black), which indicates that the DNA is essentially in the wrapping state. The RMSD values in the MD simulations of the H2A.B NCP (red) are significantly larger than those in the H2A NCP with an average value of 11.0 ± 3.0 Å. The DNA starts to unwrap from the histone octamer after 200 ns. The results suggest that the DNA in the H2A.B NCP is more dynamic than that in the H2A NCP. We also carried out the same analysis to the CG trajectories, which show significantly larger conformational changes than the MD simulations. In the CG simulations of the H2A.B NCP, the DNA is unwrapping to a larger extent than that in the CG simulations of the H2A NCP (Additional file 1: Figure S1). In the latter, the DNA is also unwrapping sometimes, which indicates an intrinsic motion in the NCP called DNA breathing [24]. The results from the CG simulations are consistent with those from the MD simulations.

To describe the DNA unwrapping more quantitatively, we calculated the distribution of the distances between the two DNA ends (the first P atom at the entry and the first P atom at the exit). Figure 2b shows the distance distribution from the MD simulations. The end-to-end distances in the H2A NCP are distributed between 55 and 90 Å with the peak value at approximately 72 Å (black). One snapshot with such a DNA end–end distance is presented (Fig. 2c), in which the DNA essentially wraps around the octamer. In the H2A.B NCP, those distances cover a significantly broader range from 55 to 150 Å with three peaks (red). The first peak is still at 72 Å that represents a wrapping state. The second peak is approximately at 95 Å and the third is around 128 Å. A representative structure indicates that one DNA end obviously unwraps but the other end remains wrapped (Fig. 2d). This asymmetric unwrapping of the DNA was also reported by other studies [25, 26]. From the CG simulations, the DNA end-to-end distances in the H2A NCP are within 45–155 Å with the peak value at 65 Å, whereas those in the H2A.B NCP are from 115 to 210 Å with the peak value at 176 Å (Additional file 1: Figure S2a). The CG simulations sample a larger structural difference between the H2A NCP (Additional file 1: Figure S2b) and the H2A.B NCP (Additional file 1: Figure S2c) than the AA-MD simulations (Fig. 2c, d). Overall, both the AA-MD and CG results suggest that the DNA-unwrapping conformations in the H2A.B NCP are much more than those in the H2A NCP.

How many base pairs of DNA are affected by H2A.B? We calculated the number of unwrapped DNA base pairs from the octamer during the MD simulations (Fig. 2e). In the H2A NCP, the average number of unwrapped base pairs is generally fewer than five (black). However, the DNA ends in the H2A.B NCP show larger fluctuations than those in the H2A NCP. There are averagely 15 base pairs that can unwrap from the octamer (red), and this number can be as large as nearly 30. Our results are in good agreement with the experimental data in that 118–130 base pairs of DNA can be protected against micrococcal nuclease digestion in the H2A.B NCP [9–11].

### Molecular mechanism of the relaxed structure in the H2A.B NCP

To investigate the molecular mechanism of DNA unwrapping from the histone octamer in the H2A.B NCP, interactions between the DNA and the histones were analyzed. A contact is defined when any pair of heavy atoms between two components is smaller than 6.0 Å. Each histone has two copies, and their contacts with DNA are averaged.

**Fig. 3:**
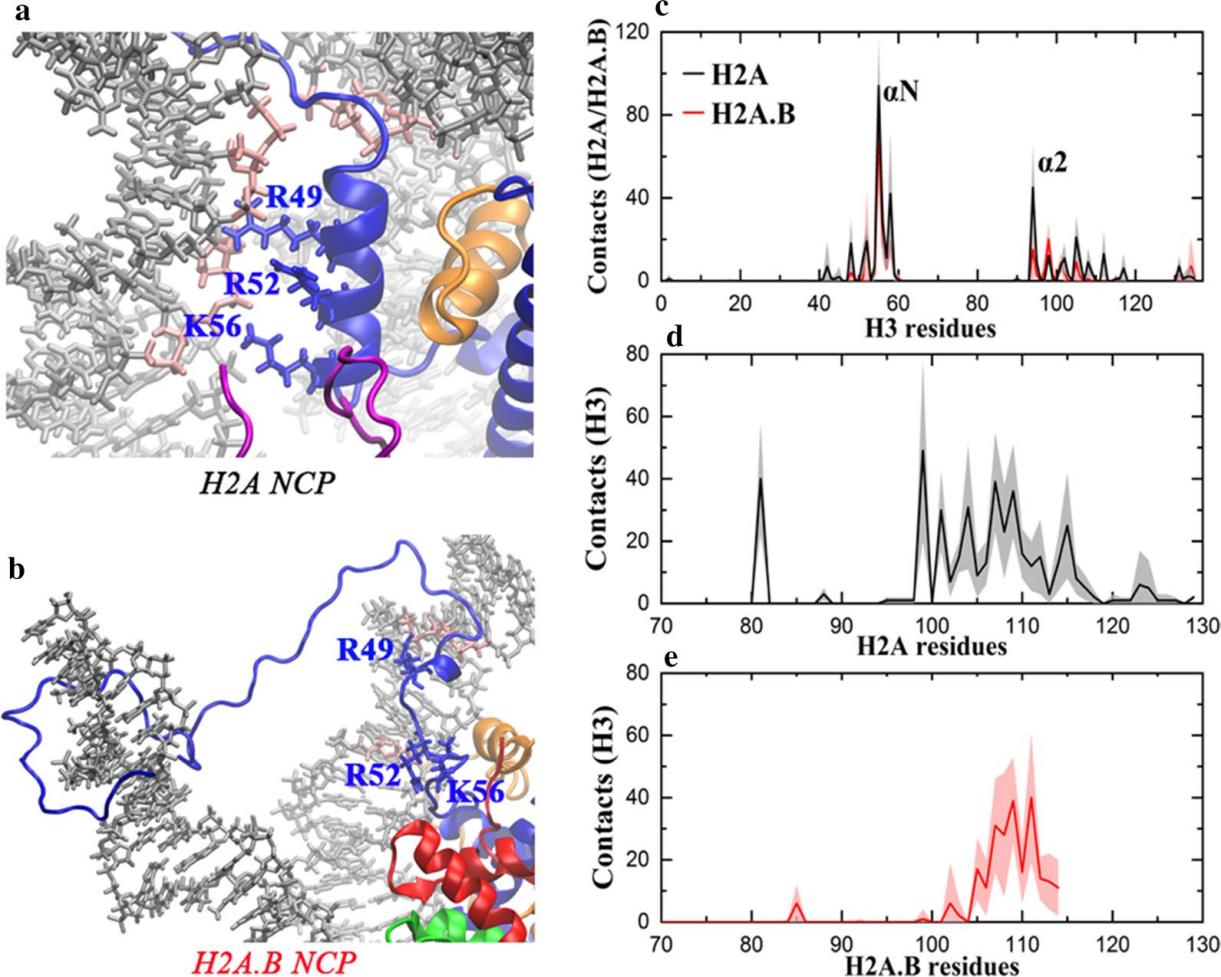
H2A.B disrupts the interactions between the H3 αN and DNA. **a** Interactions between the H3 αN and DNA in the H2A NCP, but **b** they are broken in the H2A.B NCP. The base pairs in contact with the H3 αN are colored in pink. **c** Average number of contacts of each residue in H3 with H2A (black) or H2A.B (red) during the AA-MD simulations. The αN and the α2 are labeled. **d** Average number of contacts of each residue in H2A with H3 during the AA-MD simulations. **e** Average number of contacts of each residue in H2A.B with H3 during the AA-MD simulations. In **c**–**e**, for each system, average values calculated from the three independent simulations are plotted, and standard deviations are shown as errors that are represented by shade. The contact numbers from the two copies of the same histone in one nucleosome structure are also averaged

It has been recognized that interactions between the H3 αN and DNA stabilize the DNA ends [24, 27]. From the MD simulations, the average number of contacts between the two components in the H2A.B NCP is 174 ± 50, which is significantly fewer than that in the H2A NCP (274 ± 28). The H3 αN can bind to both the ends and the inner segments of DNA in the H2A NCP, but residues like R49, R52 and K56 mainly interact with the DNA ends (Fig. 3a). However, these contacts between the three residues and the DNA ends are broken in the H2A.B NCP (Fig. 3b). This may be one of the reasons for the unwrapping of the DNA ends from the histone octamer.

How does H2A.B affect the interactions between the H3 αN and DNA? Through the analysis of the MD data, contacts between H2A.B and H3 (237 ± 41) are also decreased compared to those between H2A and H3 (332 ± 63). In particular, some broken contacts come from those with the H3 αN (Fig. 3c, 56 ± 20 in the H2A.B NCP versus 156 ± 37 in the H2A NCP). Only the docking domain and the Ct in H2A interact with H3 at the same side (Fig. 3d). However, H2A.B lacks the Ct and its docking domain is incomplete, which may lead to a weakened interaction between H2A.B and H3 (Fig. 3e). The RMSDs of the H3 αN in the MD simulations of the H2A NCP and the H2A.B NCP were calculated. The H3 αN in the H2A NCP is stable, but it has significantly larger conformational changes in the H2A.B NCP (Additional file 1: Figure S3). These results demonstrate that the lack of the Ct and part of the docking domain in H2A.B would weaken the interactions between H2A.B and H3. As a result, the H3 αN becomes mobile and its interactions with DNA are reduced as well, which make it easier for the DNA ends to unwrap from the histone octamer.

**Fig. 4:**
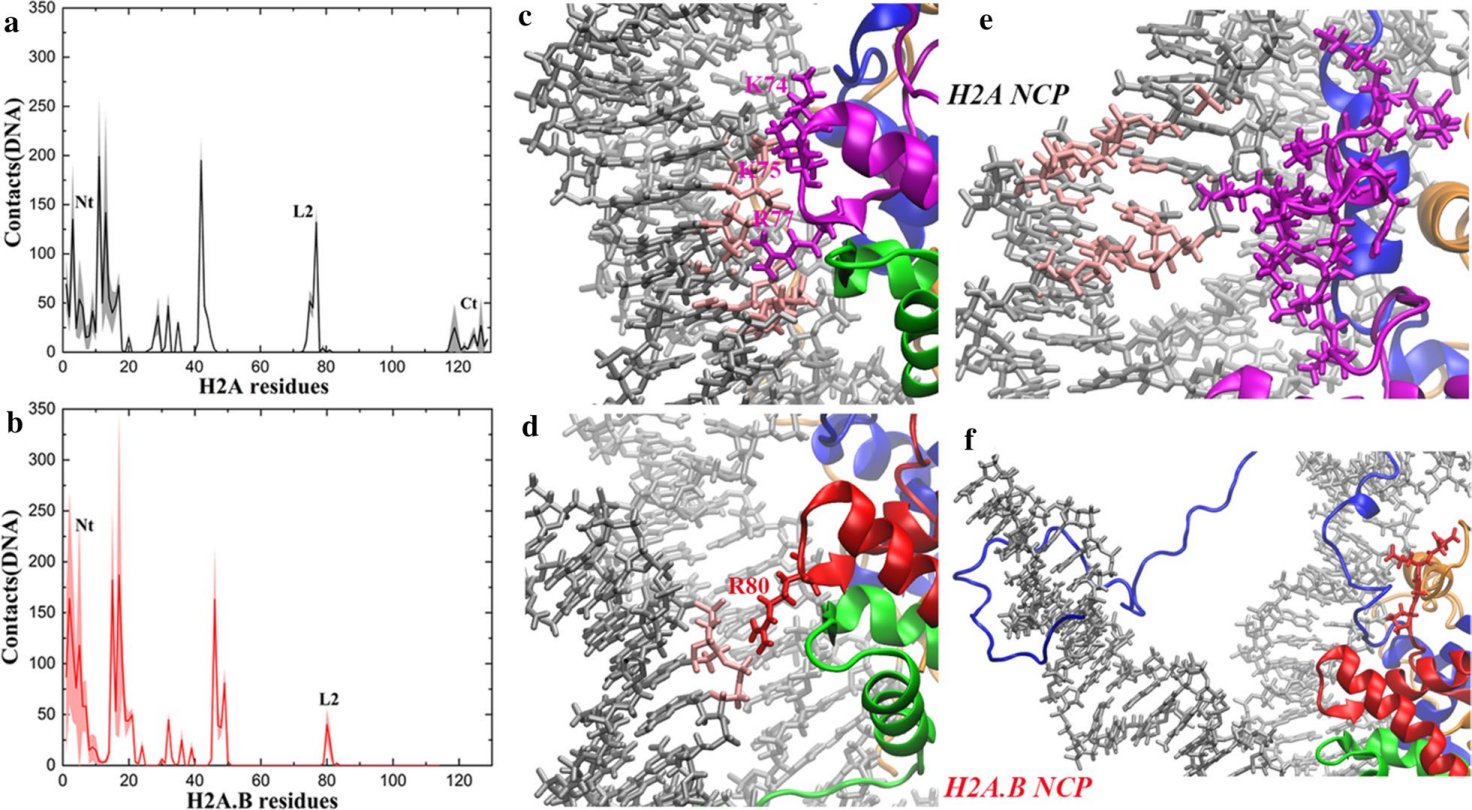
The L2 and the missing C-terminal tail in H2A.B are also responsible for the DNA unwrapping. **a** Average number of contacts of each residue in H2A with the DNA during the AA-MD simulations. The Nt, the L2 and the Ct are labeled. **b** Average number of contacts of each residue in H2A.B with the DNA during the AA-MD simulations. The Nt and the L2 are labeled. In **a**, **b**, for each system, average values calculated from the three independent simulations are plotted, and standard deviations are shown as errors that are represented by shade. The contact numbers from the two copies of the same histone in one nucleosome structure are also averaged. **c** Detailed interactions between the L2 and DNA in the H2A NCP. The base pairs in contact with the H2A L2 are colored in pink. **d** The interactions are significantly decreased in the H2A.B NCP. The base pairs in contact with the H2A.B L2 are colored in pink. **e** The H2A Ct has interactions with the DNA end in the H2A NCP, but **f** H2A.B has no Ct. The base pairs in contact with the Ct are colored in pink

It is known that the H3 αN is only responsible for the organization of approximately 13 base pairs of DNA [27]. However, our simulation results reveal that more base pairs can be detached from the histone octamer in the H2A.B NCP (Fig. 2e). Therefore, the unwrapping of the DNA in the H2A.B NCP should also be related to other features. In addition to the Ct and the docking domain, there are some other sequence differences between H2A.B and H2A. Contact analysis (Fig. 4a, b) suggests that interactions between the H2A.B L2 and DNA (63 ± 27) decrease significantly compared with those between the H2A L2 and DNA (282 ± 28). The H2A L2 has a sequence of 74-KKTRII while the H2A.B L2 has a sequence of 78-GERNII (Fig. 1a). It can be seen that the two consecutive basic residues (KK) in the H2A L2 become neutral and acidic residues (GE) in the H2A.B L2. K74, K75 and R80 in the H2A L2 can form salt bridges with the DNA (Fig. 4c), which has an important effect on the stability of the DNA. However, only R80 in the H2A.B L2 interacts with the DNA (Fig. 4d). Therefore, we believe that the weakening of the interactions between the H2A.B L2 and DNA is responsible for the detachment of more than 13 base pairs in the H2A.B NCP. In addition, the H2A Ct has a small number of contacts with DNA, as shown in Fig. 4a. From the snapshots, we see that there are partial interactions between the H2A Ct and the DNA ends (Fig. 4e), which are lost because H2A.B has no Ct (Fig. 4f). Therefore, the absence of the Ct in H2A.B may also make a minor contribution to its special properties.

**Fig. 5:**
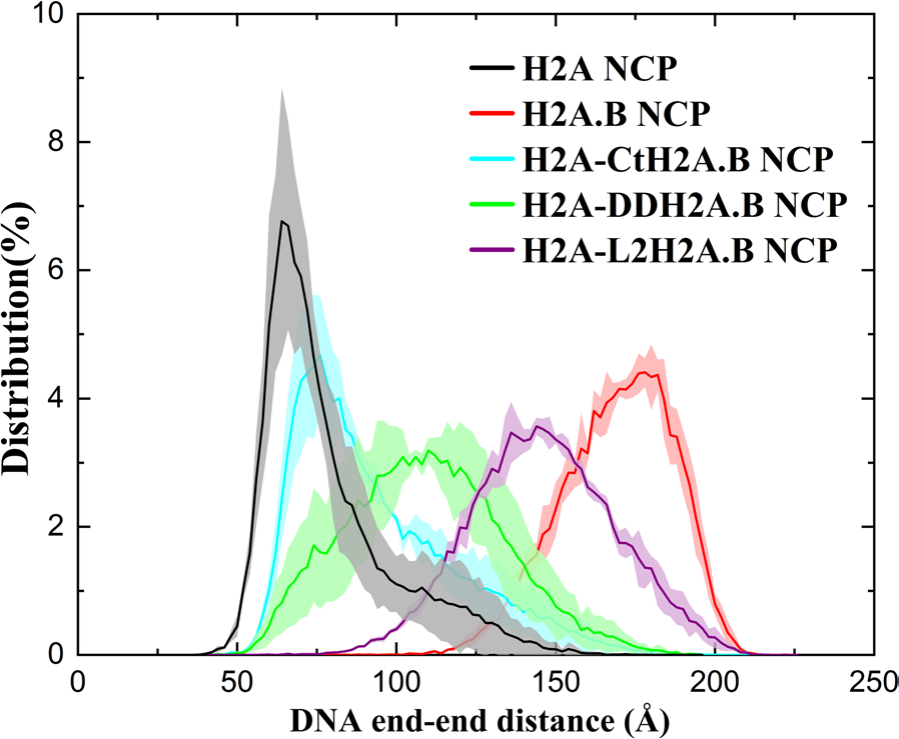
Distributions of the DNA end-to-end distances from the CG simulations of the H2A NCP, several mutated H2A NCP, and the H2A.B NCP. For each system, average values calculated from three independent CG simulations are plotted, and standard deviations are shown as errors that are represented by shade

To verify the above molecular mechanism and compare the contributions of the Ct, the docking domain, and the L2, we constructed several mutated systems including the H2A NCP with the Ct (residues 107–129) replaced by residues 109–114 of H2A.B (denoted as the H2A–CtH2A.B NCP), the H2A NCP with the docking domain and the Ct (residues 80–129) replaced by residues 84–114 of H2A.B (the H2A–DDH2A.B NCP), and the H2A NCP with the L2 loop, the docking domain and the Ct (residues 74–129) replaced by residues 78–114 of H2A.B (the H2A–L2H2A.B NCP). CG simulations were conducted and the DNA end-to-end distances were then calculated (Fig. 5). Compared to the H2A NCP (black), the distances between the two DNA ends in the H2A–CtH2A.B NCP are increased a little (cyan). After also replacing the docking domain, the distances in the H2A–DDH2A.B NCP (green) are increased more than those in the H2A–CtH2A.B NCP. If the docking domain and the L2 are both replaced, the distance distribution (purple) is pretty close to that of the H2A.B NCP (red). The results indicate that the docking domain and the L2 may contribute more to the DNA unwrapping in the H2A.B NCP than the Ct only, which is consistent with the experimental data [9]. Therefore, the DNA unwrapping in the H2A.B NCP should be caused by both the L2 and the incomplete docking domain in H2A.B.

### The H2A.B Nt may prevent further unwrapping of DNA

**Fig. 6:**
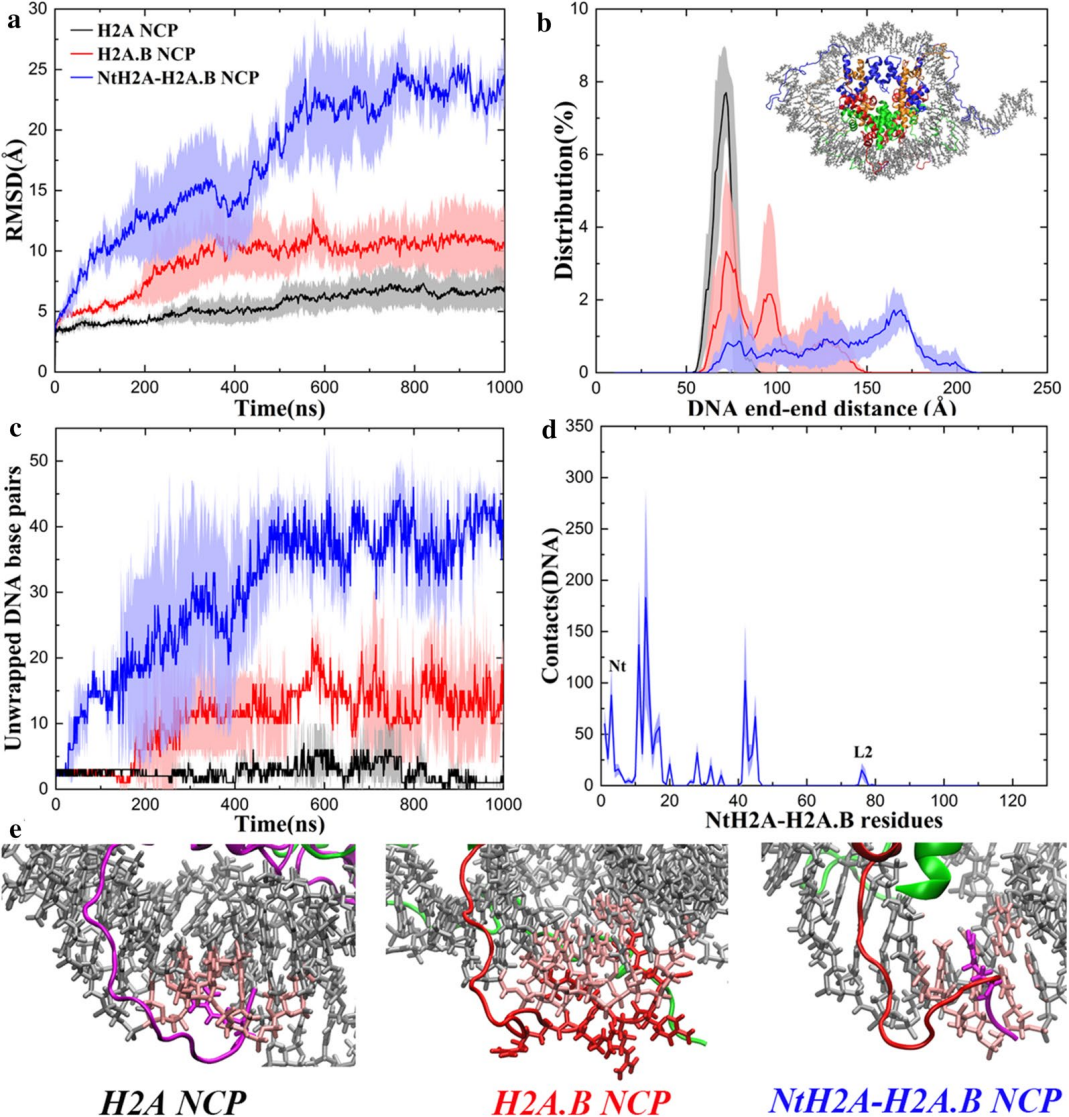
The H2A.B NCP may be even more dynamic without its Nt. **a** Time evolution of the RMSD of the DNA during the AA-MD simulations of the NtH2A–H2A.B NCP (blue). The RMSD curves of the H2A NCP (black) and the H2A.B NCP (red) are also shown for comparison. **b** Distribution of the DNA end-to-end distances from the AA-MD simulations of the NtH2A–H2A.B NCP (blue). The distributions of the H2A NCP (black) and the H2A.B NCP (red) are also shown for comparison. A representative structure of the NtH2A–H2A.B NCP that has a peak DNA end-to-end distance of 168 Å is shown in the panel. **c** Time evolution of the number of the unwrapped DNA base pairs during the AA-MD simulations of the NtH2A–H2A.B NCP (blue). The curves of the H2A NCP (black) and the H2A.B NCP (red) are also shown for comparison. **d** Average number of contacts of each residue in NtH2A–H2A.B with the DNA during the AA-MD simulations. The Nt and the L2 are labeled. In **a**–**d** for each system, average values calculated from the three independent simulations are plotted, and standard deviations are plotted as errors that are represented by shade. In **d** the contact numbers from the two copies of the same histone in one nucleosome structure are also averaged. **e** Detailed interactions between the Nt and DNA in the H2A NCP, the H2A.B NCP, and the NtH2A–H2A.B NCP. Args in the Nt are drawn in licorice. The base pairs in contact with the Nt are colored in pink

The H2A.B Nt contains a continuous stretch of six arginines, which is very different from the H2A Nt (Fig. 1a). This sequence is quite unique, and, to the best of our knowledge, there has been no relevant report on its possible biological function yet. To tackle this issue, we replaced the first eight residues of H2A.B with the first four residues of H2A, and then conducted an MD simulation for this system. Interestingly, it has been found that the DNA in the NtH2A–H2A.B NCP has an even larger conformational change than that in the H2A.B NCP. During the MD simulations, the RMSD of the DNA can be larger than 25.0 Å with an average about 23.0 ± 2.0 Å (Fig. 6a, blue), whereas those values in the H2A.B NCP are essentially smaller than 15.0 Å (Fig. 6a, red). The DNA end-to-end distances in the NtH2A–H2A.B NCP are broadly distributed from 62 to 208 Å (Fig. 6b, blue). The population of the wrapping state in the NtH2A–H2A.B NCP is very low. The peak value of the DNA end-to-end distance is approximately at 168 Å. A representative structure suggests that more base pairs in the NtH2A–H2A.B NCP may be detached from the octamer than those in the H2A.B NCP (Fig. 2d). During the MD simulations, the number of unwrapped based pairs can be more than 40 (Fig. 6c, blue).

According to the contact analysis (Fig. 4a, b), although the H2A.B L2 has weaker interactions with the DNA than the H2A L2, the H2A.B Nt has more contacts with the DNA (1362 ± 181) than the H2A Nt (1040 ± 131). This is not surprising due to these positively charged arginines in the H2A.B Nt, and such a compensation effect may allow the DNA to maintain a proper degree of unwrapping in the H2A.B NCP. However, in the NtH2A–H2A.B NCP (Fig. 6d), both the Nt and the L2 have weaker interactions with the DNA (797 ± 94 and 27 ± 38, respectively) than those in the H2A.B NCP (1362 ± 181 and 63 ± 27, respectively), which may allow more base pairs of DNA to unwrap. Therefore, we speculate that the six consecutive arginines in the Nt may play a “balancing” role in the structural dynamics of the H2A.B NCP, which compensate for the weakening interactions between the L2, the docking domain, the Ct and the DNA ends by increasing the contacts between the Nt and DNA (Fig. 6e).

### H2A.B affects the nucleosome assembly

After determining that the DNA ends in the H2A.B NCP are highly dynamic and are more likely to disassociate from the histone octamer than the H2A NCP, the effect of H2A.B on the assembly of the NCP was investigated using CG simulations.

**Fig. 7:**
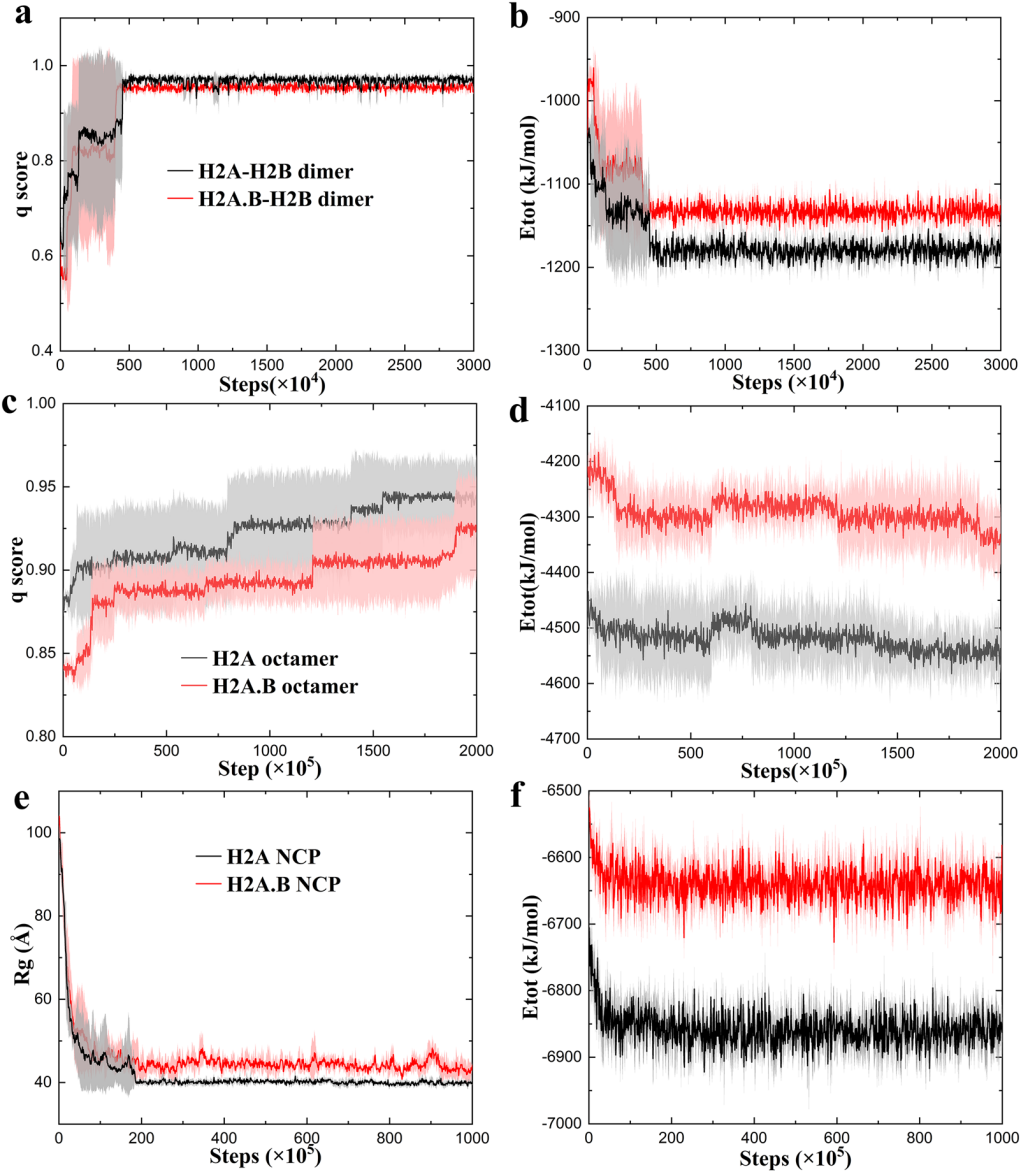
H2A.B affects the nucleosome assembly. Time evolution of the **a** q score and **b** total energy during the CG assembly of the H2A–H2B dimer (black) and the H2A.B–H2B dimer (red). Time evolution of the **c** q score and **d** total energy during the CG assembly of the H2A octamer (black) and the H2A.B octamer (red). Time evolution of the **e** Rg and **f** total energy during the DNA wrapping of the H2A NCP (black) and the H2A.B NCP (red). For each system, average values calculated from three independent CG simulations are plotted, and standard deviations are plotted as errors that are represented by shade

First, we studied the assembly of the H2A/H2A.B–H2B dimer, and each CG simulation was run for 3 × 10^7^ time steps. The q factor of a conformation is defined by the number of native contacts formed in the conformation divided by the total number of native contacts. The larger the q factor is, the more stable the system. The H2A.B–H2B dimer has generally smaller q factors (Fig. 7a) and higher energies (Fig. 7b) than the H2A–H2B dimer, which indicate that both H2A and H2A.B may form dimers with H2B, but the H2A.B–H2B dimer is less stable than the H2A–H2B dimer. At the level of the histone octamer, we set the initial state as a (H3–H4)_2_ tetramer and two H2A/H2A.B–H2B dimers, and each CG simulation was run for 2 × 10^8^ time steps. There is an intermediate in the octamer assembly, which forms a hexasome [28] containing one (H3–H4)_2_ tetramer and one H2A/H2A.B–H2B dimer. The H2A.B octamer has essentially smaller q factors (Fig. 7c) and higher energies (Fig. 7d) than the H2A octamer. The results suggest that the H2A.B octamer is also less stable than the H2A octamer, which may allow the H2A.B–H2B dimer to easily dissociate from the NCP, and thereby increase the efficiency of H2A.B in promoting transcription.

The DNA wrapping process was also investigated, and each CG simulation was run for 1 × 10^8^ time steps. Figure 7e shows the time evolution of the radius of gyration (R_g_) during the assembly. It can be found that, in both the H2A and H2A.B NCP, the R_g_ decreases sharply at the beginning and then reaches an equilibrium, which represents the process of DNA wrapping. In the H2A NCP, the average R_g_ after the assembly is about 40.0 ± 0.7 Å (Fig. 7e, black). However, in the H2A.B NCP, the wrapped DNA is still quite mobile with a larger average R_g_ of 45.0 ± 2.0 Å (Fig. 7e, red). The energy of the assembled H2A.B NCP is higher than that of the H2A NCP (Fig. 7f). Combined with the above results, it may be more difficult to assemble the H2A.B NCP than the H2A NCP.

### H2A.B may affect the structural dynamics of the chromatosome and the fiber

In addition to these effects on the NCP stability and assembly, the structural dynamics of the higher-order chromatin structures that are affected by H2A.B is also very interesting, but it has rarely been studied so far. Zhou et al. [29] have shown that H2A.B lacks an acidic patch that is crucial for the folding of the chromatin fiber. This effect has something to do with interactions at the interface between tetranucleosomal units [30], which is out of the scope of this work. Shukla et al. have suggested that the incomplete docking domain of H2A.B may disrupt the H1 binding [31]. We therefore address this issue by conducting CG simulations.

The chromatosome studied in this paper contains an NCP, two 20-bp linker DNA, and a link histone H1. How would H2A.B affect the interactions between H1 and the NCP in the chromatosome? We carried out CG simulations with 1.5 × 10^8^ time steps each for the chromatosome.

**Fig. 8:**
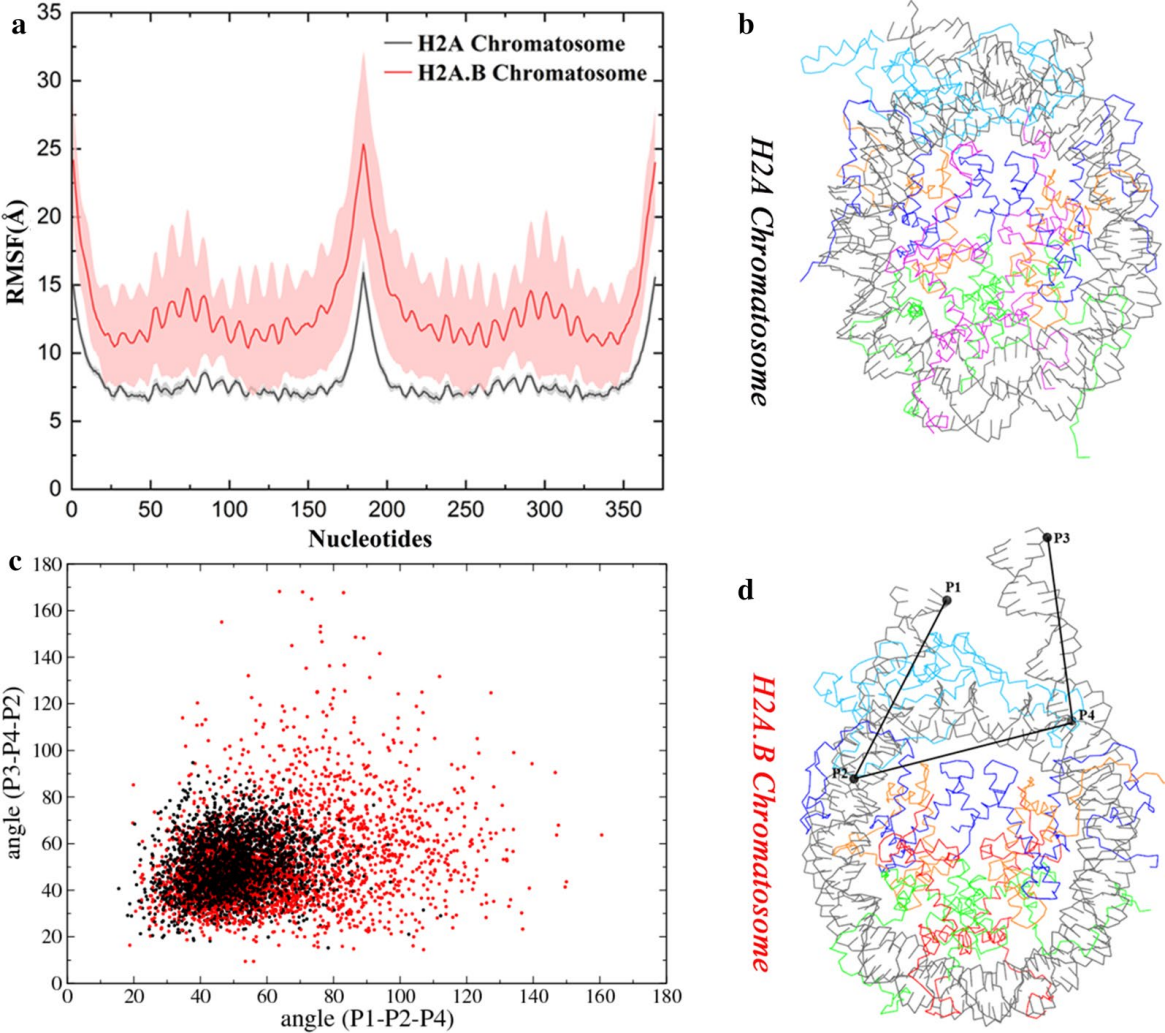
H2A.B may alter the structural dynamics of the linker DNA in the chromatosome. **a** RMSFs of the nucleotides calculated from the CG simulations. For each system, average values calculated from three independent CG simulations are plotted, and standard deviations are shown as errors that are represented by shade. **b** A snapshot of the H2A chromatosome in the CG simulation. **c** A snapshot of the H2A.B chromatosome in the CG simulation. Two angles, P1–P2–P4 and P3–P4–P2, are defined. **d** The angle distribution in the H2A chromatosome (black) and the H2A.B chromatosome (red) during the CG simulations

From the RMSFs of the DNA (Fig. 8a), one can see that the DNA in the H2A.B chromatosome (red) is looser than that in the H2A chromatosome (black). The two linker DNAs in the H2A chromatosome are generally compact and cross each other (Fig. 8b) [32], whereas in the H2A.B chromatosome, they are more extended and sometimes could be nearly parallel to each other (Fig. 8c). To show the distribution of the linker DNA, we selected two end P atoms (denoted as P1 and P2) in one linker DNA and two end P atoms (denoted as P3 and P4) in the other (shown in Fig. 8c). Two angles, P1–P2–P4 and P3–P4–P2, were then measured from the CG trajectories. These angles in the H2A.B chromatosome can adopt a broader range than those in the H2A chromatosome (Fig. 8d). The deletion of the C-terminus and the incomplete docking domain may lead to a reduced number of contacts between H2A.B and H1 [31], and thus weaken the interactions between H1 and the linker DNAs.

**Fig. 9:**
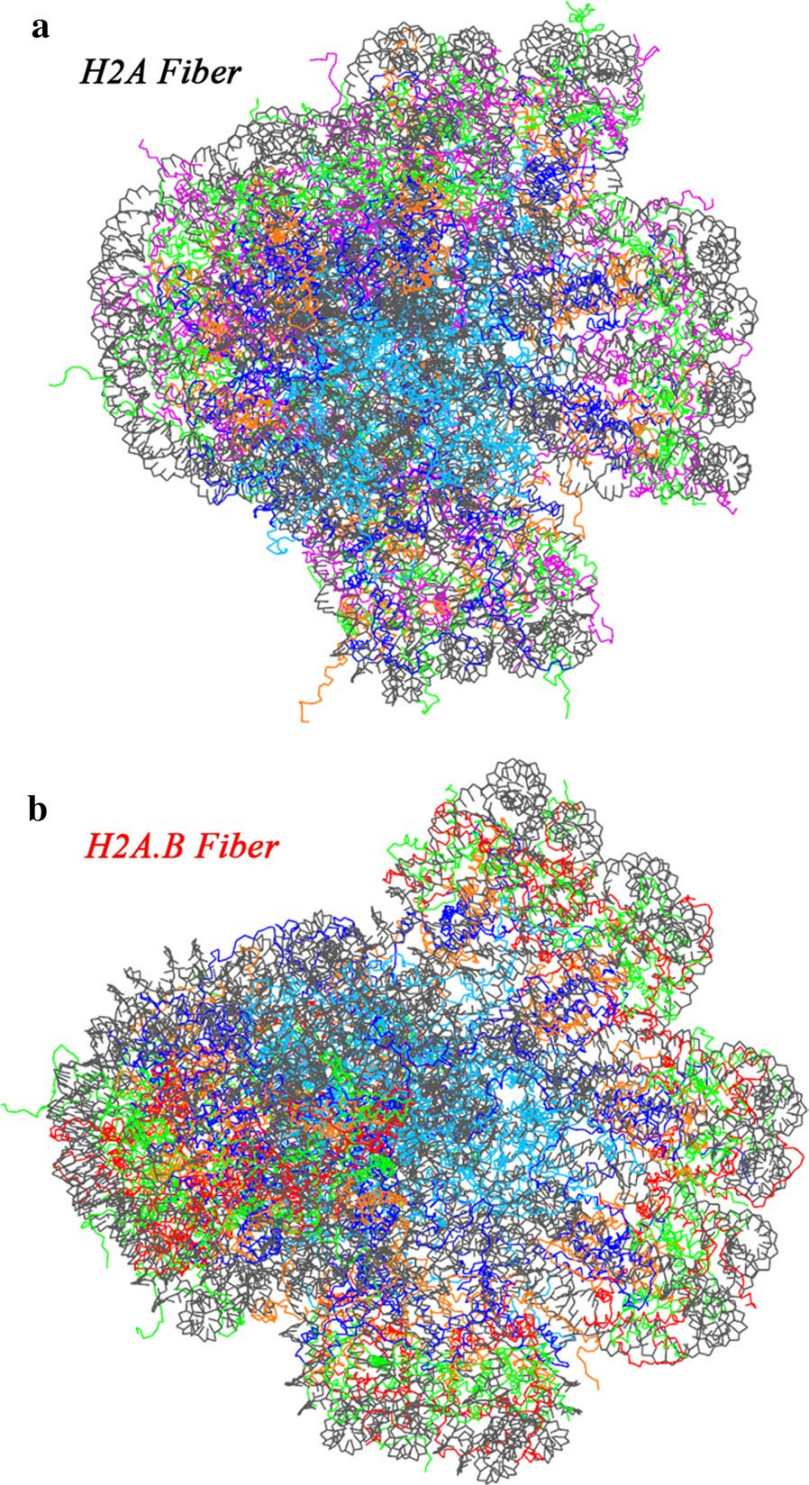
H2A.B may disrupt the structure of the chromatin fiber. **a** The final structure of the H2A fiber after the CG simulation. **b** The final structure of the H2A.B fiber after the CG simulation

It has been recognized that the compact configuration of the linker DNA induced by H1 (Fig. 8b) would favor the formation of the fiber [32]. We took a structural model of the 30-nm fiber consisting of 12 subunits of the chromatosome [33] to start CG simulations with 2 × 10^7^ time steps each. The simulation data show that the average R_g_ of the H2A fiber is 100.6 ± 0.4  Å, whereas this value for the H2A.B fiber is 102.8 ± 0.6 Å. The H2A fiber is a compact structure (Fig. 9a), but after replacing the H2A with H2A.B, the fiber becomes open (Fig. 9b). Although the discussion on existence or absence of the 30 nm fiber is still active [34–36], our preliminary simulations indicate that the extended linker DNA in the H2A.B chromatosome may disrupt the structure of the 30-nm fiber.

## Discussion

Due to the instability and large size of the H2A.B NCP, it may be difficult to solve its high-resolution structure. By using computer simulations, we are able to construct an atomic model of the H2A.B NCP and simulate its dynamic properties. In this paper, a detailed molecular mechanism of the instability of the H2A.B NCP is presented.

There are significant differences in the amino acid sequences between H2A.B and H2A. The first is the lack of the C-terminus in H2A.B, including the C-terminal tail and the last segment of the docking domain. It has been reported that the C-terminus of H2A may play an important role in regulating the chromatin structure and its dynamics [31, 37, 38], which, according to our findings, could be the result of less stable H3 αN–DNA interactions. In the H2A NCP, the C-terminal tail interacts with the entry/exit sites of DNA through hydrogen bonds, and the last segment of the docking domain has extensive interactions with the H3 αN. Therefore, the lack of the C-terminal tail might destabilize the DNA at the entry/exit sites, while the incomplete docking domain destabilizes the H3 αN. Since the H3 αN is responsible for wrapping approximately 13 base pairs of DNA at the both ends, the lack of the C-terminus in H2A.B may destabilize them. Another difference is that the L2 loop in H2A.B has less basic residues than that in H2A. The H2A L2 interacts with the DNA extensively through hydrogen bonds and salt bridges, whereas in the H2A.B NCP, these interactions are weakened dramatically because the amino acids in the H2A.B L2 are less basic with only one arginine. Therefore, these changes in the L2 loop may unwrap more base pairs in the H2A.B NCP. The aforementioned sequence changes in H2A.B could in all result in a highly dynamic structure of the H2A.B NCP, which is consistent with some experimental data [9–11]. A recent work [39] has shown that CENP-C binding to the H2A C-terminal tail can destabilize the latter and increase unwrapping of the CENP-A nucleosome, which is consistent with our study. Both the absence of the C-terminal tail in H2A.B and the destabilization of the H2A C-terminal tail by an extrinsic factor may lead to the DNA unwrapping.

Additionally, there are some other differences between the sequences of H2A.B and H2A, such as the presence of six continuous arginines in the N-terminal tail of the former. However, to the best of our knowledge, there has been no report on this issue yet. After replacing the N-terminal tail of H2A.B with that of H2A, it is very interesting to find that the NtH2A–H2A.B NCP is even more dynamic than the H2A.B NCP, and more DNA could unwrap from the histone core. These arginines in the N-terminal tail of H2A.B make extensive contacts with the DNA, which may serve as compensation to prevent the further unwrapping of the DNA in the H2A.B NCP. However, how this is related to the function of H2A.B remains puzzling.

As far as we know, there are few experimental data on the H2A.B chromatosome and fiber. Due to their large sizes, it would be very computationally expensive to study their dynamic properties using AA-MD simulations. Currently, CG simulations may be appropriate for such systems. Although the model is relatively simple, our CG simulations agree with some experimental results. A future work would be to simulate the assembly processes of the chromatosome and the fiber in the presence of H2A.B.

There are many other histone variants that play various functional roles in regulating nucleosome dynamics. The multiscale computational method used in this paper may be universally applied to study their dynamic properties. With the integration of computer simulations and more advanced experimental techniques, more details about H2A.B and other histone variants will be uncovered.

## Materials and methods

### Starting structures of the simulated systems

We first built two simulated systems of the human NCP, the H2A NCP and the H2A.B NCP, by homology modeling using MODELLER [23]. The crystal structure of the human NCP (pdb entry 3AFA) [40] does not have histone tails, but a crystal structure of the Xenopus laevis NCP (pdb entry 1KX5) [41] contains all the tails. Since the sequence identities between human and Xenopus laevis histones are 92.1% for H2A, 93.6% for H2B, 98.5% for H3 and 100% for H4, we used the two structures as templates to build an atomic model of the H2A NCP (Fig. 1b). The sequence identity between H2A and H2A.B is approximately 48%, and a structural model of the H2A.B monomer was built. The structure shows a conserved histone fold as the H2A monomer that is fairly consistent with the experimental structure [13]. The two copies of H2A in NCP were then replaced by H2A.B to obtain a structural model of H2A.B NCP (Fig. 1b).

These mutated H2A NCP and H2A.B NCP systems, including the H2A–CtH2A.B NCP, the H2A–DDH2A.B NCP, the H2A–L2H2A.B NCP, and the NtH2A–H2A.B NCP, were built by UCSF-Chimera [42].

For higher-order structures, the atomic models of the H2A chromatosome and the H2A chromatin fiber were built based on cryo-electron microscopy reconstructions [33]. H2A was then replaced by H2A.B to build a model of the H2A.B chromatosome, and a model of the H2A.B chromatin fiber, respectively.

### AA-MD simulations

Starting from the atomic model of the H2A NCP, AA-MD simulations were carried out with a parallel implementation of the GROMACS-4.5.5 package [43] using the Amber03ws force field [44]. The periodic boundary condition with a dodecahedron box type was used with a distance of 1.2 nm between the solute and the box boundary, and the TIP4P/2005 [45] water molecules were added into the box. The steepest descent method was used for the energy minimization of the system until the maximum force on any atom was smaller than 1000 kJ mol^−1^ nm^−1^. To compensate the net charge of the solute and mimic the physiological salt concentration (150 mM), K^+^ and Cl^−^ ions were added to the system by replacing water molecules with the most favorable electrostatic potential. The final system was energy minimized using the steepest descent followed by the conjugate gradient method until the maximum force on any atom was no larger than 400 kJ mol^−1^ nm^−1^. The leap-frog algorithm [46] was used with a 2-fs time-step, and an equilibration simulation of 100 ps with a positional restraint using a force constant of 1000 kJ mol^−1^ nm^−2^ was performed. The initial atomic velocities were generated according to a Maxwell distribution at 310 K. The production run was 1 us long after the system was set up and the parameters were described as follows. The simulation was performed in a constant NPT ensemble, and the system was coupled to a temperature bath of 310 K using a velocity rescaling thermostat [47]. The pressure was adjusted to 1 bar with a relaxation time of 0.5 ps, and the compressibility was 4.5 × 10^−5^ bar^−1^ [48]. Covalent bonds were constrained using the P-LINCS algorithm [49]. The twin-range cutoff distances for the van der Waals interactions were chosen to be 0.9 and 1.4 nm, and the neighbor list was updated every 20 fs. The long-range electrostatic interactions were treated by the PME algorithm [50] with a tolerance of 10^−5^ and an interpolation order of 4. AA-MD simulations were also conducted for the H2A.B NCP and the NtH2A–H2A.B NCP. Those parameters were nearly the same as the above. For each system, we conducted three independent 1-µs MD simulations.

### CG simulations

Due to the simplified potential energy and reduced number of degrees of freedom in CG models, we can observe much larger conformational changes than those in the AA-MD simulations. All CG simulations were run using the CafeMol 3.0 package [51]. For the proteins, the AICG2 + model [52] was used, which is a Go-like model with each residue represented by a CG particle located at its C_α_-atom position. Additionally, the Debye–Hückel equation was applied to calculate the electrostatic interaction. For DNA, we used a CG model called 3SPN.2C [53], in which each nucleotide is represented by three particles: one for the sugar, one for the phosphate group and one for the base. The DNA potential consists of structure-based local energy, base-stacking energy, base-pairing energy, excluded volume, and electrostatic interaction. The phosphate charge in the 3SPN.2C model is − 0.6*e*. For the potential between the protein and DNA, only the excluded volume and the Debye–Hückel-type electrostatic interactions were defined [54]. For the former, residue-type-dependent radii for both amino acids and nucleotides were used. For the latter, it should be noted that the phosphate charge was set to − 1.0*e*.

For every NCP system, three independent CG simulations with different initial velocities were carried out using Langevin dynamics at a constant temperature of 310 K. To calculate the electrostatic interactions, the point charge model was used with an ionic strength of 0.15 M. We also carried out assembly simulations for the H2A/H2A.B–H2B dimers, the H2A/H2A.B octamers, and the H2A/H2A.B NCPs. To begin the assembly simulations, some components were first separated away, and the system was centered in a cubic box with a size of 150 Å for the dimers, 300 Å for the octamers, and 500 Å for the NCPs. For every system, three independent assembly simulations with different initial positions or velocities were conducted. The structures of the H2A/H2A.B chromatosomes and the H2A/H2A.B fibers were investigated by CG simulations as well.

### Analysis

All the analysis was done using tools in the GROMACS-4.5.5 package [43]. VMD [55] was used for visualization. The program for computing the unwrapped based pairs was written by us. For DNA at either the entry or exit site of the NCP, the number of unwrapped base pairs, *N*, is determined if the minimum distance of the *N* base pairs to the histone core is larger than 6.0 Å while that of the *N *+ 1 base pair is smaller than 6.0 Å. Only heavy atoms are used to calculate distances. The total number of the unwrapped base pairs is then obtained by adding up those at the both sites.

## Supplementary information

**Additional file 1: Figure S1.** Time evolution of the RMSD of the DNA during the CG simulations of the H2A NCP and the H2A.B NCP. **Figure S2.** The H2A.B NCP is more dynamic than the H2A NCP in the CG simulations. (**a**) Distribution of the DNAs’ end-to-end distances. (**b**) A representative CG structure of the H2A NCP that has a peak DNA end-to-end distance of 65 Å. (**c**) A representative CG structure of the H2A.B NCP that has a peak DNA end-to-end distance of 176 Å. **Figure S3.** RMSDs of the H3 αN in the MD simulations of the H2A NCP (black) and the H2A.B NCP (red).

## Data Availability

Not applicable.
