# Supplementary material for: Molecular mechanism of histone variant H2A.B on stability and assembly of nucleosome and chromatin structures
Source: Epigenetics Chromatin. 2020 Jul 14;13:28. doi: 10.1186/s13072-020-00351-x (PMC7362417; doi:10.1186/s13072-020-00351-x)
Supplement: Supplementary file 1 — Additional file 1: Figure S1. Time evolution of the RMSD of the DNA during the CG simulations of the H2A NCP and the H2A.B NCP. Figure S2. The H2A.B NCP is more dynamic than the H2A NCP in the CG simulations. (a) Distribution of the DNAs’ end-to-end distances. (b) A representative CG structure of the H2A NCP that has a peak DNA end-to-end distance of 65 Å. (c) A representative CG structure of the H2A.B NCP that has a peak DNA end-to-end distance of 176 Å. Figure S3. RMSDs of the H3 αN in the MD simulations of the H2A NCP (black) and the H2A.B NCP (red). [file 13072_2020_351_MOESM1_ESM.docx]

Additonal Information

Molecular mechanism of histone variant H2A.B on stability and assembly of nucleosome and chromatin structures

Junhui Peng^1,2,#^, Chuang Yuan^1,#^, Xinfan Hua^1^, Zhiyong Zhang^1,*^

^1^MOE Key Laboratory for Membraneless Organelles & Cellular Dynamics, National Science Center for Physical Sciences at Microscale, School of Life Sciences, University of Science and Technology of China, Hefei, Anhui 230026, People’s Republic of China

^2^Present address: Laboratory of Evolutionary Genetics and Genomics, The Rockefeller University, New York, NY 10065, United States of America

^#^The authors contributed equally to this work

^*^Corresponding author: Zhiyong Zhang, Tel: +86-551-63600854; Email: zzyzhang@ustc.edu.cn


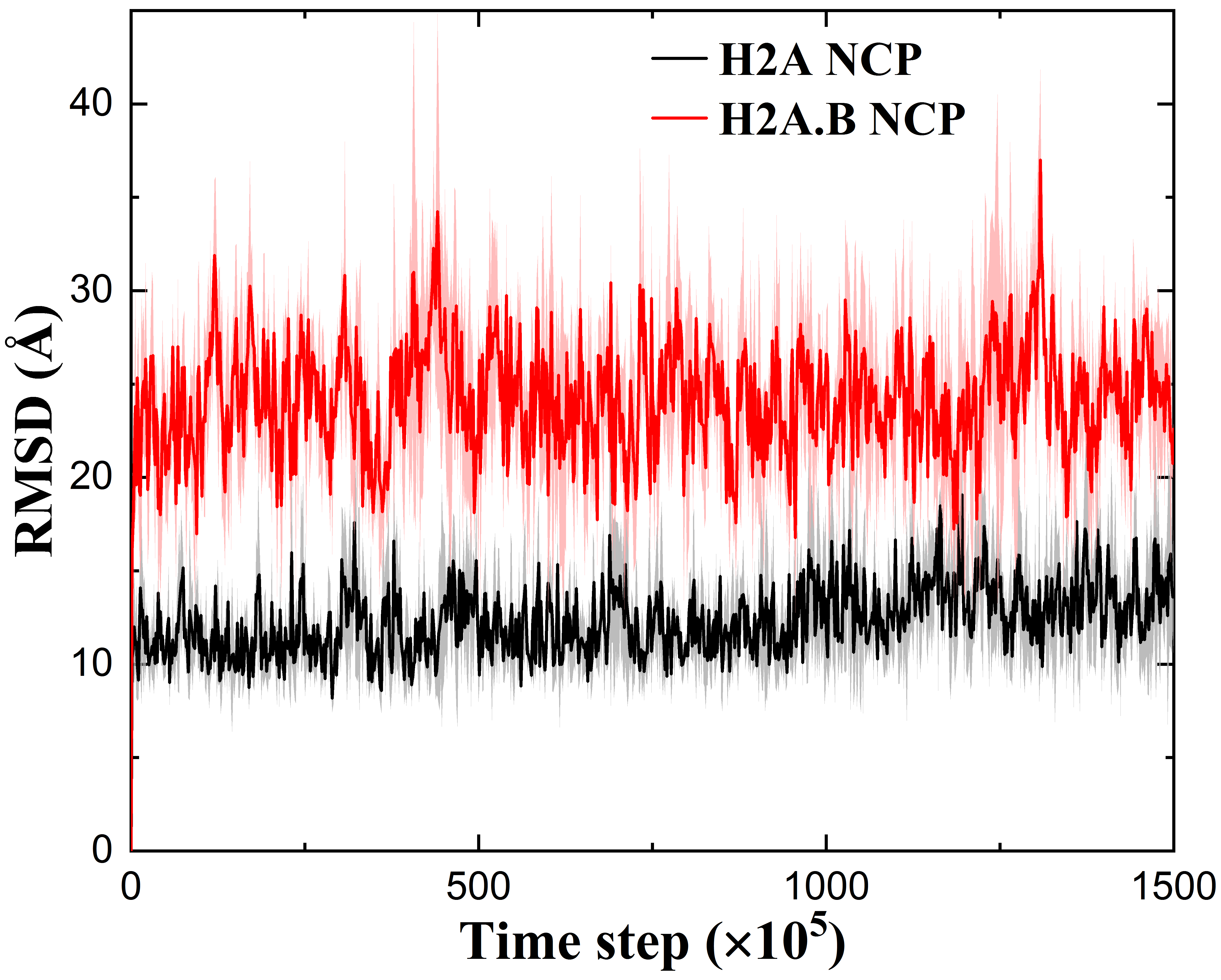


**Figure S1**. Time evolution of RMSD of the DNA during the CG simulations of the H2A NCP and the H2A.B NCP. The RMSD values were calculated using all the P atoms. For each system, average values calculated from three independent CG simulations are plotted, and standard deviations are shown as errors that are represented by shade.


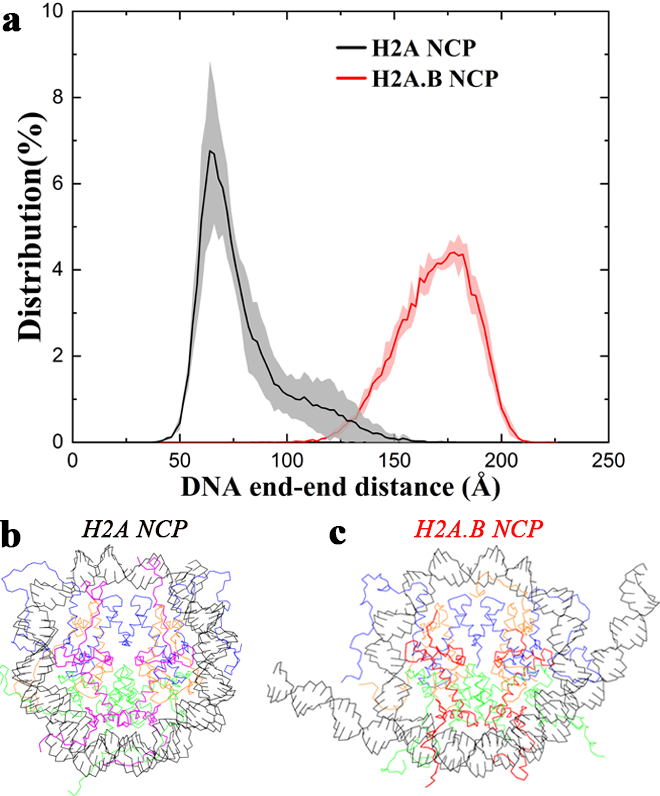


**Figure S2**. The H2A.B NCP is more dynamic than the H2A NCP in the CG simulations. (a) Distribution of the DNA end-to-end distances, which were calculated between the first P atom at the entry and the first P atom at the exit. For each system, average values calculated from three independent CG simulations are plotted, and standard deviations are shown as errors that are represented by shade. (b) A representative CG structure of the H2A NCP that has a peak DNA end-to-end distance of 65 Å. (c) A representative CG structure of the H2A.B NCP that has a peak DNA end-to-end distance of 176 Å.


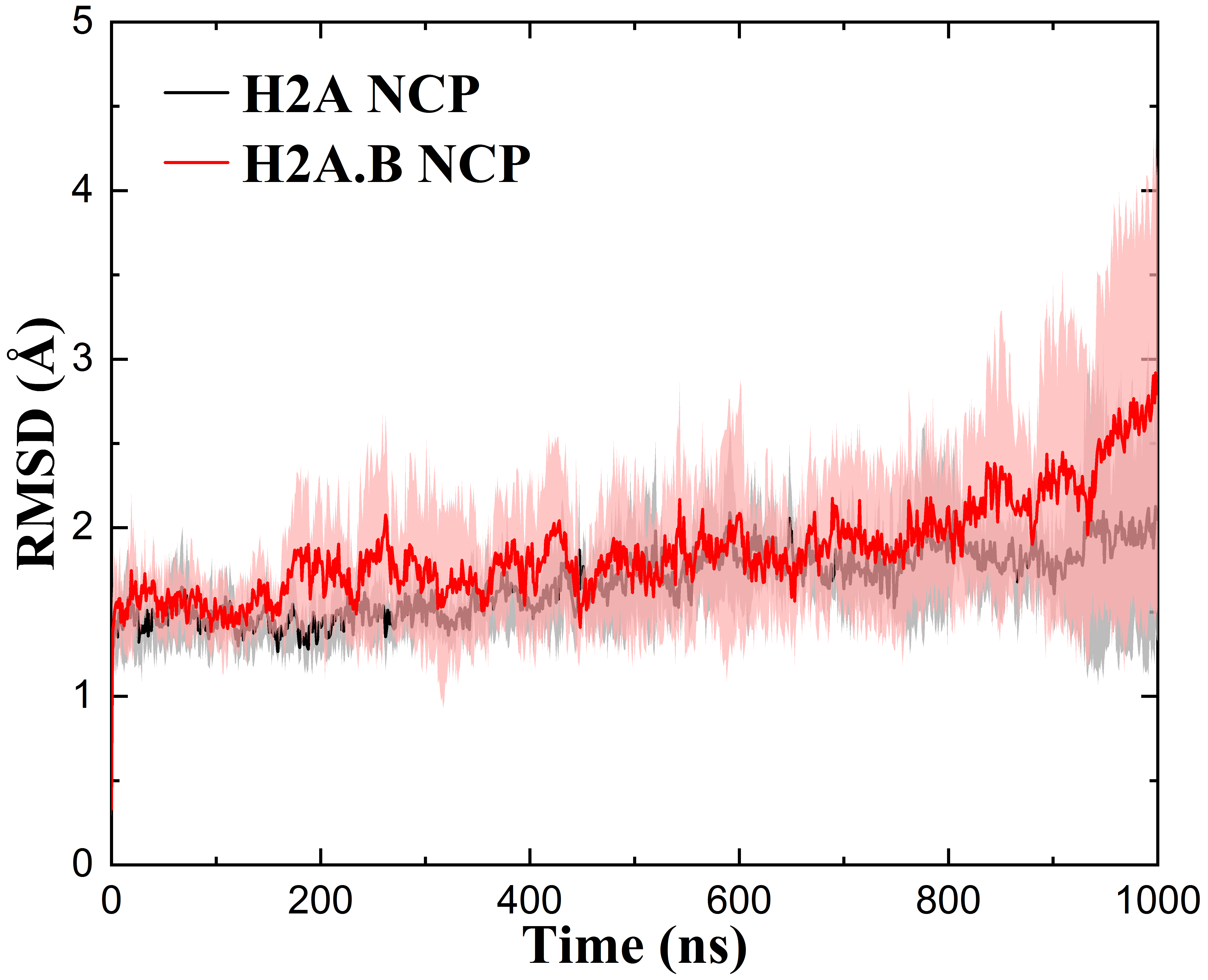


**Figure S3**. RMSDs of the H3 αN in the MD simulations of the H2A NCP (black) and the H2A.B NCP (red). The RMSD values are calculated using the heavy atoms. For each system, average values calculated from the two copies of H3 αN and three independent MD simulations are plotted, and standard deviations are shown as errors that are represented by shade.
